# Routine Laboratory Markers as Incremental Predictors Beyond OSTA for Dual-Energy X-Ray Absorptiometry-Defined Osteoporosis: Internal Validation in a Referral Cohort

**DOI:** 10.3390/diagnostics16131956

**Published:** 2026-06-23

**Authors:** Ömer Faruk Öz, Can Dinç, Özge Berfin Babayiğit, Diba Saygılı Öz, Selen Doğan, Nasuh Utku Doğan, Murat Özekinci, İnanç Mendilcioğlu

**Affiliations:** 1 Department of Obstetrics and Gynecology, Akdeniz University Faculty of Medicine, 07070 Antalya, Türkiye; 2 Department of Radiology, Antalya City Hospital, Kepez, 07080 Antalya, Türkiye

**Keywords:** postmenopausal osteoporosis, dual-energy X-ray absorptiometry, uric acid, systemic immune-inflammation index, OSTA, prediction model

## Abstract

**Background and Objectives:** Routine laboratory markers may support diagnostic risk stratification for osteoporosis, but their incremental value beyond the Osteoporosis Self-Assessment Tool for Asians (OSTA) remains uncertain in referral-based practice. We evaluated whether serum uric acid, albumin, alkaline phosphatase (ALP), and systemic inflammatory indices improve prediction of DXA-defined osteoporosis beyond OSTA in postmenopausal women. **Materials and Methods:** This retrospective cross-sectional study included 3504 postmenopausal women referred for DXA between January 2021 and May 2025. Osteoporosis was defined as the lowest T-score ≤ −2.5 at the lumbar spine, total hip, or femoral neck. Sequential exclusions removed patients with chronic hepatobiliary disease, chronic systemic inflammatory disease, bone-active medication exposure, systemic glucocorticoid use, abnormal liver biochemistry, or missing required variables. Multivariable logistic regression assessed associations, and OSTA-based prediction models were internally validated using stratified 10-fold cross-validation. **Results:** Osteoporosis was present in 1660 women (47.4%). Higher BMI, uric acid, and albumin were independently associated with lower odds of osteoporosis, whereas ALP and calcium were associated with higher odds. OSTA alone achieved an AUC of 0.679. Adding uric acid, albumin, and ALP increased AUC to 0.695 and slightly improved the Brier score, with good calibration. Adding the systemic immune-inflammation index did not materially improve performance. **Conclusions:** Routine laboratory variables provided only modest incremental value beyond OSTA. The model should be interpreted as an exploratory referral-pathway prioritization approach, not as a standalone population-screening tool. It should not be used as a diagnostic surrogate for DXA or as a fracture-risk model.

## 1. Introduction

Postmenopausal osteoporosis is a major public health problem because fragility fractures are associated with pain, disability, loss of independence, and increased mortality, especially after hip fracture [1,2,3]. Dual-energy X-ray absorptiometry (DXA) remains the reference standard for defining osteoporosis using T-scores [4,5], but access to DXA and structured fracture-risk assessment is often limited in high-volume clinical settings. Practical tools that can prioritize DXA referral are therefore clinically relevant, provided that their intended use is clearly distinguished from population-wide screening or treatment decision-making. Contemporary densitometry positions also emphasize central DXA sites for diagnosis while reserving the 33% radius for selected circumstances [6].

FRAX integrates clinical risk factors, with or without BMD, to estimate fracture probability [7]. In contrast, OSTA is a simpler tool based only on age and body weight [8]. Its simplicity is attractive for electronic health record workflows, but it does not capture biochemical or inflammatory states that may reflect bone turnover, nutritional reserve, renal handling, or systemic health. Postmenopausal bone loss is biologically linked to estrogen deficiency, osteoclast activation, cytokine signaling, the RANK/RANKL/OPG axis, and oxidative stress [9,10,11,12]. These mechanisms provide a rationale for studying readily available markers such as serum uric acid, albumin, ALP, and complete blood count-derived inflammatory indices.

Serum uric acid is a major contributor to plasma antioxidant capacity but is also linked to adiposity, renal handling, diet, and metabolic health [13,14]. Prior studies in postmenopausal women have reported heterogeneous associations between uric acid, bone mineral density, bone turnover, and vertebral fracture [15,16,17,18]. Systemic inflammatory indices such as SII, NLR, PLR, and MLR are inexpensive and widely available, and several studies have linked them to BMD or osteoporosis risk [19,20,21]. We aimed to evaluate the associations of serum uric acid and systemic inflammatory indices with DXA-defined osteoporosis and to assess whether routine laboratory variables provide incremental predictive value beyond OSTA in a DXA-referred cohort of postmenopausal women. The intended use was DXA triage/referral prioritization rather than diagnosis replacement, treatment decision-making, or fracture-risk estimation.

## 2. Materials and Methods

### 2.1. Study Design and Setting

This retrospective cross-sectional study used a de-identified electronic health record/DXA analysis extract of postmenopausal women referred for and undergoing DXA at Akdeniz University Hospital between January 2021 and May 2025. The study was reported in accordance with STROBE and TRIPOD guidance for observational and prediction model reporting [22,23]. Because this was a DXA-referred clinical cohort rather than a population-based screening sample, all model performance estimates were interpreted as internal evidence for diagnostic referral-pathway prioritization within a referral-based setting.

Ethical approval was obtained from Akdeniz University Medical Scientific Research Ethics Committee (TBAEK-863). Because de-identified retrospective data were used, informed consent was waived according to institutional policy.

### 2.2. Participants and Exclusion Criteria

Eligible participants were postmenopausal women with available DXA-derived T-scores and routine laboratory data. During the study period, 7687 DXA examinations were identified. After removing 1140 repeat DXA examinations from the same patients, 6547 unique patients remained. Sequential exclusions were applied for patients who did not meet the female-sex eligibility criterion (*n* = 2591), non-postmenopausal status or unavailable menopausal-status information (*n* = 119), missing valid lumbar spine, total hip, or femoral neck T-score data (*n* = 38), missing demographic data required for age, weight, BMI, or OSTA calculation (*n* = 45), chronic hepatobiliary disease (*n* = 33), chronic systemic inflammatory disease (*n* = 71), antiresorptive therapy use (*n* = 86), hormone replacement therapy/SERM use (*n* = 13), systemic glucocorticoid use (*n* = 7), abnormal liver biochemistry (*n* = 12), and missing required routine laboratory variables (*n* = 28). The final cohort included 3504 unique postmenopausal women. Exclusion criteria were applied sequentially, and patients meeting more than one criterion were counted only at the first applicable exclusion step. The participant flow is shown in Supplementary Table S1.

To reduce confounding from non-skeletal ALP sources, chronic systemic inflammation, and treatment-related phenotypic masking, the source EHR query excluded patients with ICD-10 evidence of chronic hepatobiliary disease or chronic systemic inflammatory disease before model development. Recorded antiresorptive therapy, hormone replacement therapy/SERM use, or systemic glucocorticoid exposure before or at the time of DXA were also exclusion criteria. Abnormal liver biochemistry was defined as ALT, AST, GGT, or total bilirubin above the institutional upper reference limit in available laboratory testing before or at the time of DXA.

### 2.3. Outcome, Predictors, and Measurements

The primary outcome was DXA-defined osteoporosis, defined as the lowest T-score ≤ −2.5 at the lumbar spine (L1–L4), total hip, or femoral neck [4,5]. Women not meeting this threshold were classified as non-osteoporotic (normal or osteopenia).

These central DXA sites were selected because they were the routinely available and clinically standardized sites in the institutional DXA database. In line with the positions of the International Society for Clinical Densitometry (ISCD), the 33% radius may be used in selected circumstances, such as non-interpretable hip or spine measurements, hyperparathyroidism, or body size exceeding table limits, whereas heel quantitative ultrasound is primarily a fracture-risk or triage modality and its T-scores should not be used interchangeably with central DXA for WHO diagnostic classification [6]. Distal radius DXA and heel measurements were not available in the analytic extract and were therefore not included in the outcome definition.

DXA examinations were performed using a Discovery QDR densitometer (Hologic, Inc., Marlborough, MA, USA), and T-scores were generated using APEX software version 3.3 (Hologic, Inc., Marlborough, MA, USA) according to the manufacturer’s standard acquisition and analysis procedures. Routine biochemical parameters, including serum uric acid, albumin, alkaline phosphatase, creatinine, and calcium, were measured as part of routine clinical care in the hospital central laboratory using a Siemens Atellica analyzer (Siemens Healthineers, Erlangen, Germany) with routine clinical reagents, calibrators, and quality-control procedures. Complete blood count parameters were measured using a Sysmex XN-1000 hematology analyzer (Sysmex Corporation, Kobe, Japan).

Demographic variables included age, BMI, and menopause duration. OSTA was calculated as (weight [kg] − age [years]) × 0.2, with the decimal part truncated toward zero, as originally described [8]. Laboratory variables included serum uric acid, albumin, ALP, creatinine, calcium, and complete blood count-derived inflammatory indices. SII was calculated as platelet count × neutrophil count/lymphocyte count; NLR, PLR, and MLR were calculated as neutrophil/lymphocyte, platelet/lymphocyte, and monocyte/lymphocyte ratios, respectively [19,20,21,24,25,26,27].

The final analytic extract did not retain exact blood sampling dates relative to DXA for the routine predictors; therefore, laboratory-DXA timing could not be standardized to a fixed window. This limitation is particularly relevant to CBC-derived inflammatory indices, which may vary with transient infection, inflammatory flares, medication exposure, and other short-term clinical conditions. Menopause duration was extracted as recorded in the clinical record; the dataset did not distinguish natural from surgical menopause.

### 2.4. Statistical Analysis

Continuous variables are presented as mean ± standard deviation when approximately normally distributed and as median (interquartile range) otherwise. Across DXA categories, age and BMI were compared using one-way analysis of variance, whereas skewed variables were compared using Kruskal–Wallis tests. Multivariable logistic regression evaluated associations with osteoporosis. ALP was scaled per 10 U/L, creatinine per 0.1 mg/dL, and SII per 100 units for interpretability. Odds ratios are reported with 95% confidence intervals.

The final cohort used for descriptive statistics and OSTA-based prediction models included 3504 women with complete data for the DXA-defined outcome, age, BMI, OSTA, uric acid, albumin, ALP, creatinine, calcium, and CBC-derived indices. Menopause duration was unavailable for 178 women (5.1%). Because menopause duration was included in the fully adjusted association model, that model used complete cases (*n* = 3326). These 178 records remained in the overall cohort and in the OSTA-based prediction models. Variable-level missingness is summarized in Supplementary Table S2.

Prediction models were fitted using (1) OSTA alone, (2) OSTA plus uric acid, albumin, and ALP, and (3) Model 2 plus SII. Because OSTA already incorporates age and body weight, age was not added again to Models 2 or 3. Internal validation used stratified 10-fold cross-validation with out-of-fold predicted probabilities. Performance was assessed by AUC, calibration intercept and slope, Brier score, and decision curve analysis across threshold probabilities of 0.05–0.60 [28,29,30]. Because hepatobiliary disease, chronic systemic inflammatory disease, bone-active medication exposure, systemic glucocorticoid use, and abnormal liver biochemistry were excluded a priori, estimates represent a restricted DXA-referred cohort rather than an unrestricted clinical population.

All statistical analyses were performed using R version 4.5.3 (R Foundation for Statistical Computing, Vienna, Austria) in RStudio version 2026.05.1+225 (Posit Software, PBC, Boston, MA, USA).

## 3. Results

### 3.1. Cohort Characteristics

The final analysis cohort included 3504 postmenopausal women. Osteoporosis was present in 1660 women (47.4%), while 438 (12.5%) had normal BMD and 1406 (40.1%) had osteopenia (Table 1). Age increased and BMI decreased across worsening DXA categories. Median menopause duration was longest in the osteoporosis group, and OSTA scores decreased stepwise across categories. Serum uric acid and albumin were slightly lower in women with osteoporosis, whereas total ALP was higher. Calcium, creatinine, and CBC-derived inflammatory indices did not show clinically large between-group differences.

Menopause duration was unavailable for 178 of 3504 women (5.1%), while all other variables summarized in Supplementary Table S2 were complete. The fully adjusted association model was therefore based on 3326 complete cases, but the OSTA-based prediction models used the full cohort.

### 3.2. Multivariable Associations with Osteoporosis

In complete-case multivariable logistic regression, lower BMI and higher ALP were independently associated with osteoporosis, whereas higher uric acid and albumin were associated with lower odds (Table 2). Calcium showed a modest positive association. Age and menopause duration were not independently associated after adjustment, and SII showed only borderline inverse association. NLR, PLR, and MLR were not included in the final model because of shared information with SII and limited incremental contribution beyond the selected predictors.

### 3.3. Prediction Model Performance

**Figure 1:**
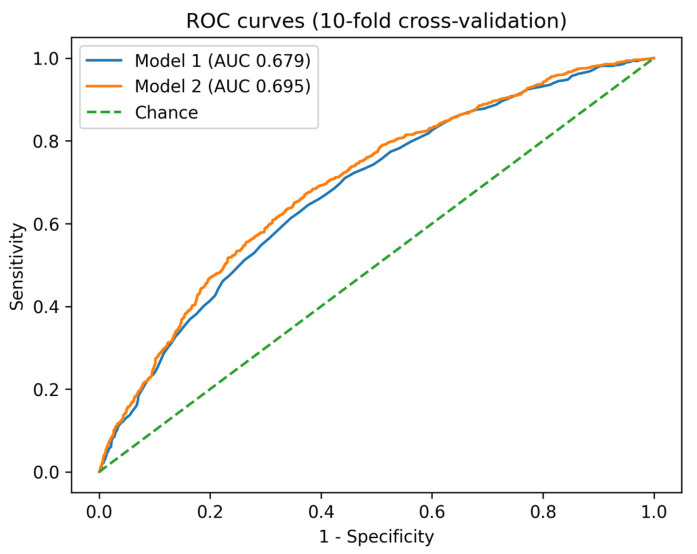
Receiver operating characteristic curves for osteoporosis prediction. ROC curves based on stratified 10-fold cross-validated out-of-fold predicted probabilities for Model 1 (OSTA alone) and Model 2 (OSTA plus uric acid, albumin, and ALP).

**Figure 2:**
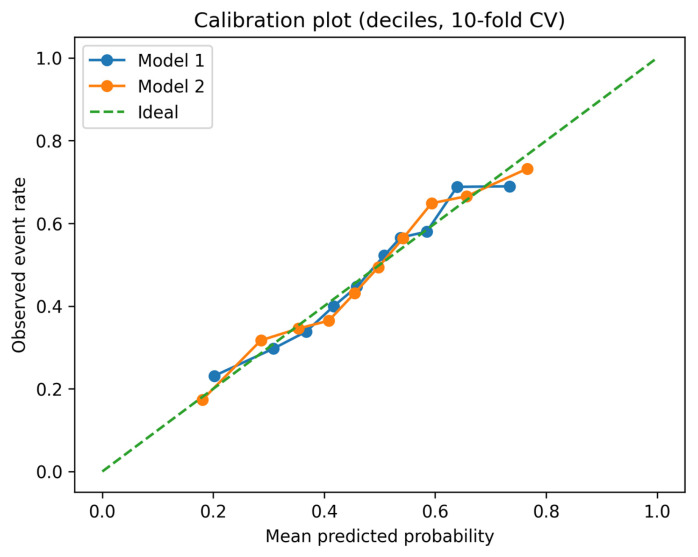
Calibration plot for osteoporosis prediction. Calibration of predicted probabilities for Model 1 and Model 2 based on stratified 10-fold cross-validated out-of-fold predictions grouped by deciles of predicted risk.

**Figure 3:**
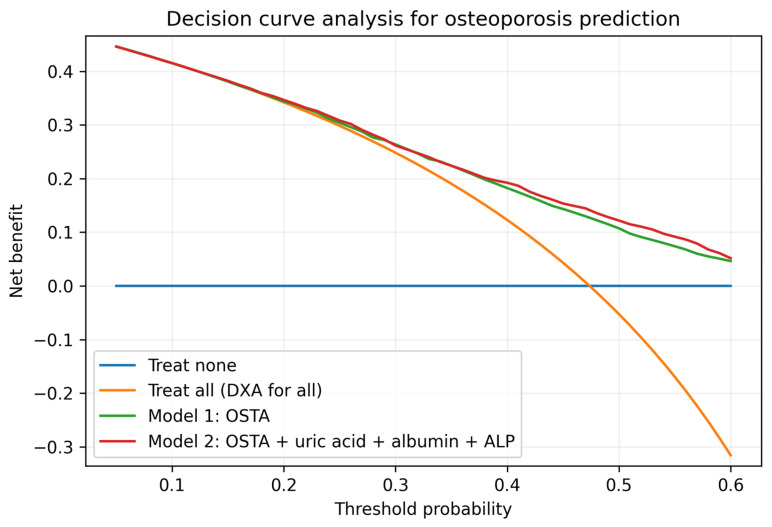
Decision curve analysis for osteoporosis prediction. Net benefit curves for Model 1 and Model 2 compared with default strategies (DXA for all and DXA for none), based on stratified 10-fold cross-validated out-of-fold predictions.

Internal validation showed modest discrimination for all models (Table 3). OSTA alone achieved an AUC of 0.679. Adding uric acid, albumin, and ALP increased the AUC to 0.695 and reduced the Brier score from 0.225 to 0.221, with good calibration. Adding SII did not materially improve discrimination or accuracy. ROC and calibration plots are shown in Figure 1 and Figure 2. Decision curve analysis showed largely overlapping net benefit curves, with only modest advantage for the laboratory-enhanced model at several intermediate thresholds (Figure 3).

The full-sample Model 2 equation was: logit(p) = 0.913 + (−0.241 × OSTA) + (−0.056 × uric acid [mg/dL]) + (−0.023 × albumin [g/L]) + (0.104 × ALP/10 [U/L]), where p is the predicted probability of osteoporosis.

## 4. Discussion

In this large DXA-referred cohort of postmenopausal women, routine laboratory variables provided statistically detectable but clinically modest incremental information beyond OSTA. Higher uric acid and albumin were associated with lower odds of DXA-defined osteoporosis, while ALP was positively associated. However, the absolute improvement in discrimination was small, and adding SII did not materially improve prediction. These findings support a cautious interpretation: routine laboratory data may help refine referral-pathway prioritization, but they do not establish a standalone screening tool. The absolute cross-validated AUC gain was 0.016 (0.679 to 0.695), and this should be viewed as a small refinement rather than a clinically decisive improvement. The model is not a diagnostic surrogate for DXA, does not estimate fracture probability, and should not guide treatment decisions without standard clinical assessment.

The uric acid finding is consistent with studies suggesting that higher uric acid may be associated with higher BMD or lower bone turnover in postmenopausal women [15,16], but the magnitude of association was modest. Uric acid is closely linked to renal handling, adiposity, diet, and metabolic health [13,14], so it should be viewed as a pragmatic risk marker rather than a bone-specific biomarker. The present results are most consistent with a multimarker approach in which uric acid contributes small amounts of information when combined with anthropometry and routine laboratories.

The ALP and albumin findings require careful interpretation. Total ALP is not bone-specific; it may reflect skeletal turnover but can also be influenced by hepatobiliary disease and other non-skeletal processes. Because recorded chronic hepatobiliary disease and abnormal liver biochemistry were excluded a priori, the observed ALP association is less likely to reflect overt hepatobiliary disease. Nevertheless, total ALP cannot be interpreted as a direct bone-turnover marker without bone-specific ALP or other turnover markers such as CTX or P1NP [31]. Albumin may capture nutritional reserve, frailty, systemic health, or inflammation, but it is also non-specific. The positive calcium association was counterintuitive and should be considered hypothesis-generating because vitamin D, parathyroid hormone, supplementation, renal/endocrine factors, and some treatment details were not systematically available.

From a cost perspective, the proposed laboratory-enhanced model is most defensible when uric acid, albumin, and ALP are already available as part of routine clinical work-up, because their marginal use in an electronic triage workflow may be low. However, ordering additional tests solely to apply this model would generate new costs, and the present study did not include a formal cost-effectiveness analysis or direct comparison with local DXA pricing. Therefore, the model should not be presented as a cost-saving alternative to DXA; any implementation would require local health-economic evaluation that accounts for test prices, reimbursement, DXA access, and downstream referral consequences.

SII did not add meaningful predictive value beyond OSTA plus laboratory variables. This may reflect the clinical composition of a DXA-referred cohort, adjustment for BMI and albumin, or measurement error. Because exact laboratory timing relative to DXA was unavailable, CBC-derived indices may have captured transient infection, inflammatory flares, or short-term medication effects rather than chronic low-grade inflammation related to bone remodeling. Such non-standardized measurement is expected mainly to attenuate associations toward the null.

Selection and spectrum bias remain central limitations. The cohort consisted only of women referred for DXA and had a high osteoporosis prevalence of 47.4%; therefore, the model should not be generalized to community screening without external validation. Calibration is prevalence-dependent, and intercept updating or broader recalibration would be required before use in lower-prevalence settings. The model was intentionally framed as referral-pathway prioritization rather than population screening or treatment guidance. Moreover, because OSTA was originally developed in Asian women, direct transferability of its thresholds to Turkish postmenopausal women should not be assumed; external validation and recalibration are required before broader use. The cohort was derived from a single tertiary referral center in Türkiye; race/ethnicity was not recorded, and the model was not tested in racially or ethnically diverse populations.

Several clinically important variables were unavailable, including prior fragility fracture, parental hip fracture or detailed family history, smoking, alcohol intake, vitamin D and parathyroid hormone status, calcium supplementation, chronic kidney disease staging, and bone turnover markers. Recorded hepatobiliary disease, chronic systemic inflammatory disease, antiresorptive therapy, HRT/SERM use, systemic glucocorticoid exposure, and abnormal liver biochemistry were addressed by a priori exclusion, reducing but not eliminating confounding. Unrecorded diagnoses, undocumented or remote medication exposure, subclinical liver dysfunction below exclusion thresholds, and inflammatory activity not captured in structured records may still influence both laboratory profiles and DXA findings. In addition, menopause duration was recorded as a single clinical variable, and natural versus surgical menopause could not be distinguished; this may leave residual heterogeneity in skeletal risk related to abrupt estrogen loss. Distal radius DXA and heel QUS/pDXA measurements were also unavailable; therefore, site-specific osteoporosis outside the lumbar spine/hip framework could not be evaluated. The exact interval between blood sampling and DXA was unavailable, so albumin, total ALP, and CBC-derived inflammatory indices may reflect nonspecific or transient clinical states rather than stable skeletal physiology.

A strength of this study is the combination of a large real-world cohort, standardized DXA-defined outcome, transparent participant flow, and internal validation with discrimination, calibration, Brier score, and decision curve analysis. Future work should externally validate the model in independent referral-based, primary-care, European, and Middle Eastern cohorts; assess calibration drift; and determine whether adding clinical risk factors, bone turnover markers, or DXA-adjacent metrics such as trabecular bone score improves clinically meaningful risk stratification [32].

## 5. Conclusions

In this DXA-referred cohort of postmenopausal women, uric acid and albumin were inversely associated with DXA-defined osteoporosis, whereas ALP was positively associated. Adding uric acid, albumin, and ALP to OSTA produced only a modest improvement in discrimination and did not yield a clinically deployable screening tool. The findings are best interpreted as exploratory support for referral-pathway prioritization and require external validation and recalibration before clinical use. Thus, the model should be regarded only as hypothesis-generating support for DXA triage in similar referral settings, not as a diagnostic substitute or fracture-risk tool.

## Figures and Tables

**Table 1:** Participant characteristics by DXA category.

Variable	Normal (T ≥ −1) (*n* = 438)	Osteopenia (−2.5 < T < −1) (*n* = 1406)	Osteoporosis (T ≤ −2.5) (*n* = 1660)	*p* Value
Age, years	54.23 ± 7.07	56.06 ± 8.44	57.94 ± 8.00	<0.001
BMI, kg/m^2^	30.24 ± 4.74	28.89 ± 5.10	27.13 ± 4.62	<0.001
Menopause duration, years	8.00 (5.00–13.00)	11.00 (6.00–17.00)	13.00 (8.00–19.00)	<0.001
OSTA score	4.00 (3.00–6.00)	3.00 (1.00–5.00)	2.00 (0.00–3.00)	<0.001
Serum uric acid, mg/dL	4.90 (4.33–5.40)	4.60 (4.10–5.30)	4.50 (3.90–5.20)	<0.001
Albumin, g/L	45.00 (43.00–46.73)	45.00 (43.00–47.00)	45.00 (43.00–46.99)	0.003
Alkaline phosphatase, U/L	75.00 (63.00–90.00)	79.00 (65.00–95.00)	83.00 (66.00–104.00)	<0.001
Creatinine, mg/dL	0.72 (0.65–0.82)	0.71 (0.64–0.80)	0.71 (0.64–0.80)	0.395
Calcium, mg/dL	9.60 (9.30–9.82)	9.60 (9.35–9.90)	9.65 (9.37–9.91)	0.067
Systemic immune-inflammation index (SII)	443.08 (326.26–637.84)	446.10 (330.41–617.88)	436.74 (318.91–601.11)	0.122
Neutrophil-to-lymphocyte ratio (NLR)	1.70 (1.30–2.34)	1.69 (1.34–2.23)	1.70 (1.33–2.20)	0.737
Platelet-to-lymphocyte ratio (PLR)	115.59 (95.90–147.10)	114.89 (91.14–141.76)	113.88 (91.61–141.51)	0.135
Monocyte-to-lymphocyte ratio (MLR)	0.22 (0.18–0.29)	0.23 (0.18–0.28)	0.23 (0.18–0.29)	0.639

**Table 2:** Multivariable logistic regression for DXA-defined osteoporosis.

Predictor	Adjusted OR (95% CI)	*p* Value
Age (per 1 year)	1.030 (0.982–1.081)	0.224
BMI (per 1 kg/m^2^)	0.899 (0.884–0.914)	<0.001
Menopause duration (per 1 year)	1.015 (0.968–1.065)	0.538
Uric acid (per 1 mg/dL)	0.928 (0.865–0.994)	0.034
Albumin (per 1 g/L)	0.966 (0.944–0.989)	0.005
ALP (per 10 U/L)	1.106 (1.076–1.136)	<0.001
Creatinine (per 0.1 mg/dL)	1.020 (0.997–1.044)	0.088
Calcium (per 1 mg/dL)	1.222 (1.036–1.442)	0.017
SII (per 100 units)	0.981 (0.961–1.000)	0.054

**Table 3:** Internal validation performance of prediction models (10-fold cross-validation).

Model	AUC (95% CI)	Brier Score	Calibration Intercept	Calibration Slope
Model 1: OSTA	0.679 (0.662–0.696)	0.225	−0.000	0.996
Model 2: OSTA + uric acid + albumin + ALP	0.695 (0.679–0.712)	0.221	−0.002	0.979
Model 3: Model 2 + SII	0.696 (0.679–0.713)	0.221	−0.003	0.977

## Data Availability

The datasets used and/or analyzed during the current study are not publicly available due to privacy and ethical restrictions but are available from the corresponding author upon reasonable request and subject to institutional approvals.

## Supplementary Materials

The following supporting information can be downloaded at https://www.mdpi.com/article/10.3390/diagnostics16131956/s1, Table S1: Participant and analytic sample flow; Table S2: Variable-level missing data summary.
