# Supplementary material for: Routine Laboratory Markers as Incremental Predictors Beyond OSTA for Dual-Energy X-Ray Absorptiometry-Defined Osteoporosis: Internal Validation in a Referral Cohort
Source: Diagnostics (Basel). 2026 Jun 23;16(13):1956. doi: 10.3390/diagnostics16131956 (PMC13359531; doi:10.3390/diagnostics16131956)
Supplement: Supplementary file 1 [file diagnostics-16-01956-s001.zip › diagnostics-4383246-supplementary.pdf]

Supplementary Materials

Manuscript title: Routine Laboratory Markers as Incremental Predictors Beyond OSTA for Dual-Energy X-ray Absorptiometry-Defined Osteoporosis: Internal Validation in a Referral Cohort

Supplementary Table S1. Participant and analytic sample flow

**Selection / analytic step**	***n***
Total DXA examinations performed between January 2021 and May 2025	7687
Excluded: repeat DXA examinations from the same patient	1140
Unique patients with DXA records	6547
Excluded: patients who did not meet the female-sex eligibility criterion	2591
Excluded: non-postmenopausal status or unavailable menopausal-status information	119
Excluded: missing valid lumbar spine, total hip, or femoral neck T-score data	38
Excluded: missing demographic data required for age, weight, BMI, or OSTA calculation	45
Excluded: chronic hepatobiliary disease	33
Excluded: chronic systemic inflammatory disease	71
Excluded: antiresorptive therapy use	86
Excluded: hormone replacement therapy or SERM use	13
Excluded: systemic glucocorticoid use	7
Excluded: abnormal liver biochemistry	12
Excluded: missing required routine laboratory variables, including serum uric acid, albumin, ALP, calcium, creatinine, or CBC-derived indices	28
Final analysis cohort	3504
Full cohort used for descriptive statistics and OSTA-based prediction models	3504
Menopause duration unavailable	178
Complete-case multivariable association model	3326

Note. Exclusion criteria were applied sequentially in the order shown. Patients meeting more than one exclusion criterion were counted only at the first applicable exclusion step. Abnormal liver biochemistry was defined as elevation of ALT, AST, GGT, or total bilirubin above the institutional upper reference limit in laboratory testing available before or at the time of DXA. The final analysis cohort included unique postmenopausal women with valid DXA outcome data and complete variables required for the OSTA-based prediction models. Menopause duration was not required for the OSTA-based prediction models but was included in the fully adjusted multivariable association model; therefore, this model was based on 3326 complete cases.

Supplementary Table S2. Variable-level missing data summary

Missingness was assessed in the final analysis dataset.

Variable / analytic component	Available *n*	Missing *n*	Missing %
DXA-defined osteoporosis outcome	3504	0	0.0
Age	3504	0	0.0
Body mass index	3504	0	0.0
Menopause duration	3326	178	5.1
OSTA score	3504	0	0.0
Serum uric acid	3504	0	0.0
Albumin	3504	0	0.0
Alkaline phosphatase	3504	0	0.0
Creatinine	3504	0	0.0
Calcium	3504	0	0.0
Systemic immune-inflammation index	3504	0	0.0
Neutrophil-to-lymphocyte ratio	3504	0	0.0
Platelet-to-lymphocyte ratio	3504	0	0.0
Monocyte-to-lymphocyte ratio	3504	0	0.0
Complete-case sample for fully adjusted model	3326	178	5.1

Note. Missing percentage was calculated as missing *n* / 3504 × 100. Menopause duration was unavailable for 178 women (5.1%); all other variables summarized in this table were complete. The complete-case multivariable regression sample was therefore 3326 women, whereas the OSTA-based prediction models used the full cohort (*n*=3504).
